# An *in vitro* simulation model to assess the severity of edge loading and wear, due to variations in component positioning in hip joint replacements

**DOI:** 10.1002/jbm.b.33991

**Published:** 2017-09-23

**Authors:** O. O'Dwyer Lancaster‐Jones, S. Williams, L. M. Jennings, J. Thompson, G. H. Isaac, J. Fisher, M. Al‐Hajjar

**Affiliations:** ^1^ Institute of Medical and Biological Engineering, School of Mechanical Engineering, University of Leeds Leeds United Kingdom; ^2^ DePuy Synthes Joint Reconstruction Leeds United Kingdom

**Keywords:** hip replacement, edge loading, surgical positioning, wear, ceramic‐on‐ceramic

## Abstract

The aim of this study was to develop a preclinical *in vitro* method to predict the occurrence and severity of edge loading condition associated with the dynamic separation of the centres of the head and cup (in the absence of impingement) for variations in surgical positioning of the cup. Specifically, this study investigated the effect of both the variations in the medial–lateral translational mismatch between the centres of the femoral head and acetabular cup and the variations in the cup inclination angles on the occurrence and magnitude of the dynamic separation, the severity of edge loading, and the wear rate of ceramic‐on‐ceramic hip replacement bearings in a multi‐station hip joint simulator during a walking gait cycle. An increased mismatch between the centres of rotation of the femoral head and acetabular cup resulted in an increased level of dynamic separation and an increase in the severity of edge loading condition which led to increased wear rate in ceramic‐on‐ceramic bearings. Additionally for a given translational mismatch, an increase in the cup inclination angle gave rise to increased dynamic separation, worst edge loading conditions, and increased wear. To reduce the occurrence and severity of edge loading, the relative positions (the mismatch) of the centres of rotation of the head and the cup should be considered alongside the rotational position of the acetabular cup. This study has considered the combination of mechanical and tribological factors for the first time in the medial–lateral axis only, involving one rotational angle (inclination) and one translational mismatch. © 2017 The Authors Journal of Biomedical Materials Research Part B: Applied Biomaterials Published by Wiley Periodicals, Inc. J Biomed Mater Res Part B: Appl Biomater, 106B: 1897–1906, 2018.

## INTRODUCTION

Hip joint replacement is considered one of the most successful orthopaedic surgeries, however, failures still occur. Revisions due to wear in hard‐on‐hard bearings and material fatigue in hard‐on‐soft bearings of hip bearings have been associated with edge loading.^1^, ^2^, ^3^, ^4^ Edge loading can be defined as the condition where the contact area between the head and the cup is located on the rim chamfer of the acetabular cup. The occurrence of edge loading is a multifactorial phenomenon. Such factors include implant surgical positioning, implant design, surgical and patient factors such as cup and stem migration after surgery, and variations in patients' anatomy, biomechanics and soft tissue tension. While edge loading is multi‐factorial, it is possible to consider two different modes, one involving impingement and lever out of the head from the cup and a second which does not involve impingement, but involves separation of the centres of the head and cup, which in the extreme may lead to subluxation. Impingement can occur in many patients and prostheses and can be associated with discrete activity. Analysis has been performed which describes the effect of rotational position of the cup, cup coverage and head size and stem design on impingement.^5^, ^6^, ^7^ Edge loading, due to separation can occur in many patients, and most importantly is common in activities such as standard walking, and as such may occur frequently in some patients.^8^, ^9^, ^10^ Separation and edge loading without impingement is the focus of this study. In previous tribological studies, it was reported that edge loading due to separation can result in increased wear in hard‐on‐hard bearings and increased deformation in polyethylene bearings.^11^, ^12^, ^13^, ^14^, ^15^, ^16^, ^17^, ^18^, ^19^, ^20^ In all these previous studies the level of dynamic separation was fixed as an input, in effect edge loading was studied with a predetermined level of severity. In this study, surgical positioning was considered as the input and the level of dynamic separation, severity of edge loading and wear as the outputs of the study.

The implant surgical position comprises the position of the femoral head and stem and the acetabular cup in six degrees of freedom. Variations in surgical positioning of the acetabular cup, for example, can be rotational (i.e., around the three axes of rotations) or translational along three axes (i.e., the medial–lateral, the anterior‐posterior and the superior‐inferior axes).^21^ In rotational positioning, a steep cup inclination angle condition could result in the contact area intersecting with the edge of the acetabular cup causing increased stress.^22^, ^23^, ^24^ The coverage angle of the acetabular cup and the direction of the load will also determine at what level of rotational positioning that edge loading will occur. Variations in translational positioning encompass the position of the head centre relative to the cup centre and joint centre. Failure to restore the centres of rotation of the femoral head and acetabular cup will result in edge loading due to microseparation conditions.^11^

Microseparation (or separation if it is greater than one millimetre) is a dynamic condition where the centres of rotation of the femoral head and the acetabular cup migrate away from each other during a proportion of the gait cycle (or any other activity). This can result in the femoral head directly contacting the rim/chamfer of the acetabular cup under loading. Fluoroscopy studies have provided the evidence of this mechanism, of dynamic separation, which has occurred consistently in a proportion of the patient population,^8^, ^25^, ^26^ and can occur frequently in standard activities such as walking. Microseparation was first described and simulated *in vitro* by Nevelos et al.,^11^ who replicated stripe wear features that had been observed on revised *in vivo* ceramic‐on‐ceramic implants. *In vitro* studies involving positioning the acetabular cup at a steep inclination angle were not successful in replicating the stripe wear features that were observed on those retrievals.^27^ Nevelos et al.^11^ applied variation in translational positioning which resulted in a controlled dynamic microseparation (0.5 mm) during the gait cycle, where the centre of rotation of the acetabular cup migrated away from the centre of rotation of the femoral head. Stripe wear was replicated on the femoral head with an associated wear area at the rim of the acetabular cup. Moreover, the size distribution of the wear debris generated under this *in vitro* method matched the bi‐modal wear debris size distribution collected from the periprosthetic tissue surrounding the revised implant with stripe wear features.^1^, ^2^, ^28^, ^29^

Many *in vitro* studies have been published studying the wear rate and effect of important parameters when the head and the cup are concentric such as the effect of protein concentration in lubricants,^30^, ^31^, ^32^ third body contamination,^33^ kinematic conditions,^34^, ^35^ lubricant temperature,^36^ load profile,^37^ and material degradation^38^, ^39^ under standard simulator conditions. The effect of edge loading on the wear rates of joint replacement bearings with different cup inclination angles has been investigated when a defined level of microseparation has been prescribed as an input.^11^, ^12^, ^13^, ^14^, ^15^, ^16^, ^17^, ^18^, ^19^, ^20^, ^27^, ^40^, ^41^, ^42^ For polyethylene acetabular cups under edge loading, the contact stress increases at the rim of the acetabular cup which could lead to excessive deformation and fatigue of the material.^23^ Simulator studies have shown that the wear of ceramic‐on‐ceramic bearings were not affected under a steep cup inclination angle of 65°.^17,^ 27 However, introducing edge loading through dynamic microseparation as a consequence of mismatch in the centres of rotation of the femoral head and acetabular cup, resulted in increased wear rates and stripe wear mechanisms seen clinically. This increase was from below 0.1 mm^3^/million cycles under standard gait to 0.13 mm^3^/million cycles for BIOLOX^®^ *delta* and 1.85 mm^3^/million cycles for BIOLOX^®^ *forte*.^11^, ^12^, ^14^, ^17^ Metal‐on‐metal bearings, on the other hand, were affected by variations in surgical positioning in both rotational and translational axes. Many studies have shown that edge loading due to steep cup inclination angle can lead to significant increase in wear rates from below 1 mm^3^/million cycles under standard condition to 1–4 mm^3^/million cycles under adverse edge loading conditions with elevated metal ion levels.^19^, ^41^, ^43^ Edge loading due to fixed level of microseparation has caused a further increase in the wear for metal‐on‐metal bearings (2–9 mm^3^/million cycles) to levels seen in some cases on failed prostheses.^16^, ^19^, ^41^ Simulator studies have also shown how variations in the acetabular metal cup design feature such as the coverage angle could lead to variations in their clinical outcome.^16^, ^19^

These previous studies were undertaken by applying and controlling a fixed level of dynamic separation displacement of approximately 0.5 mm between the centres of the femoral head and acetabular cup which allowed direct comparisons between different materials under such a fixed adverse condition. However, they did not investigate the effect of cup positioning on the level of dynamic separation and severity of edge loading. Clinically, both the likelihood and the level of separation between the centres of the femoral head and acetabular cup during an activity will be affected by a number of clinical, patient and design variables, including the level of initial surgical mismatch between the centres of rotation of the femoral head and acetabular cup and combinations with other factors such as the orientation of the acetabular cup, soft tissue tension and the biomechanics of the patient. Hence it is necessary to define the surgical positioning as the input to the study and the level of dynamic separation, severity of edge loading and wear and deformation as outputs. The aim of this study was to develop a more realistic preclinical *in vitro* method to predict the occurrence and severity of edge loading condition associated with variations in surgical positioning. It was also aimed to determine the effect of the variations in the medial–lateral surgical translational mismatch between the centres of the femoral head and acetabular cup and variations in the cup inclination angles on the occurrence and magnitude of the dynamic separation, the severity of edge loading, and the wear rate of ceramic‐on‐ceramic hip replacement bearings.

## MATERIALS AND METHODS

The hip replacement bearings used in this study were 36 mm diameter BIOLOX^®^ *delta* (zirconia platelets toughened alumina) ceramic‐on‐ceramic bearings (DePuy Synthes Joint Reconstruction, Leeds, UK) where the liner was inserted into a titanium metal shell (Pinnacle^®^, DePuy Synthes Joint Reconstruction, Leeds, UK) and the head was locked onto a stainless steel stem with a 12/14 taper (C‐Stem^®^ AMT, DePuy Synthes Joint Reconstruction, Leeds, UK). The ceramic‐on‐ceramic bearings had a nominal diametrical clearance of 100 µm. The acetabular shell was cemented into a metallic cup holder at the desired inclination angle and the femoral stem was cemented vertically into a metallic holder with 20° of anteversion. The Leeds II Anatomical Physiological Hip Joint Simulator was used in this study. It is a six‐station machine capable of applying two independently controlled axes of rotations and dynamic axial load.

**Figure 1:**
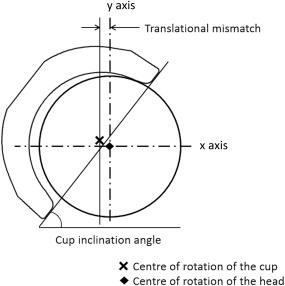
Schematic showing the acetabular cup positioned at a particular inclination angle and translational mismatch.

This study was conducted in three stages. The first stage was a biomechanical test aimed to determine the magnitude of the medial–lateral dynamic separation between the femoral head and the acetabular cup and the magnitude of the load reached under edge loading as a result of variations in component positioning in the medial–lateral axis, including acetabular cup inclination angle (rotational positioning) and level of mismatch between the centres of rotation of the femoral head and acetabular cup (translational positioning) illustrated in Figure 1. The second stage was another biomechanical study to further assess the severity of edge loading for specific conditions. The third stage aimed to assess the wear of ceramic‐on‐ceramic bearings under different levels of severity of edge loading due to variations in component positions. For all stages, a standard simulator gait cycle^34^ was used which comprises twin peak vertical load (70 N swing phase load and 2.5 kN peak load), flexion–extension (+30° to −15°) and internal–external rotation (±10°). The lubricant used was 25% new‐born calf serum (protein concentration of 15 g/L) supplemented with 0.03% of sodium azide to retard bacterial growth. For the wear study stage, the serum was changed every approximately 330,000 cycles.

**Figure 2:**
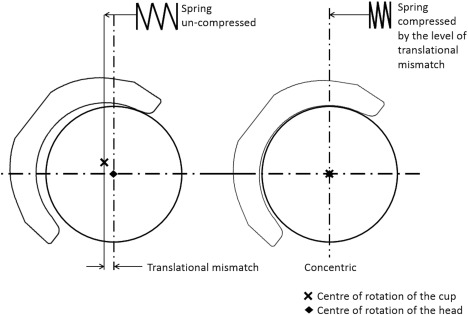
Schematic illustrating the state of the spring when the head and cup are separated (left) and during concentric conditions (right).

The biomechanical testing (stage 1) was completed using one station of the Leeds II hip joint simulator. Three different acetabular cup inclination angles were considered. These were equivalent to *in vivo* angles of 45°, 55°, and 65°.^41^ The 45° cup inclination angle is currently considered a target inclination angle during surgery whereas 55° and 65° angle are considered steep cup inclination angles. Four different levels of translational mismatches between the centres of rotation of the femoral head and acetabular cup were considered, a medial displacement of the centre of acetabular cup of 1, 2, 3, and 4 (mm) relative to the centre of the femoral head. This resulted in 12 different combinations and each condition was run for a total of 1000 cycles and repeated three times. In order to represent translational mismatch the femoral head and acetabular cup were set up concentrically on the machine, then the cup was moved medially away from the femoral head by the desired mismatch amount (1, 2, 3, or 4 mm); this introduced a superior translation of the acetabular cup as well as a medial translation due to the spherical shape of the acetabular cup. A spring with a spring constant of 100 N/mm was then positioned horizontally in the medial–lateral axis to hold the cup in position at the desired translational mismatch. This resulted in the femoral head and acetabular cup being concentric when high vertical load was applied and the horizontal spring was compressed by the same amount as the initial medial–lateral mismatch in component centres used as an input (Figure 2).

**Figure 3:**
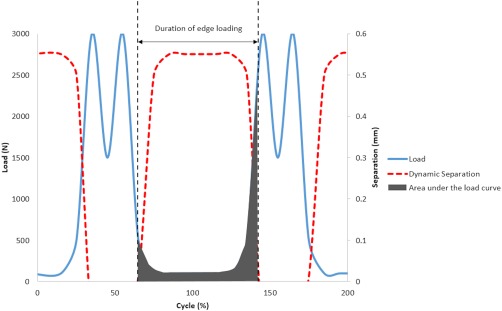
A graph showing a typical load ‘F(y)’ and dynamic separation output indicating the magnitude of dynamic separation, the duration of edge loading and area under load curve to determine the severity of edge loading condition.

The measurements (outputs) recorded in the biomechanical study were the dynamic separation displacement between the centres of the femoral head and acetabular cup during the gait cycle; the maximum vertical load at 0.1 mm of medial–lateral separation between the centres of rotation of the femoral head and the acetabular cup during relocation, and the severity of edge loading condition [(1)), Figure 3]. The vertical dynamic load was measured using a 4.5 kN load cell, the medial–lateral load measured using a 1 kN load cell and, the medial–lateral displacement of the acetabular cup was measured using a linear voltage differential transducer, LVDT. LabView software was used to acquire the data simultaneously from the load cell and LVDT. The displacement, maximum load at the rim, and severity of edge loading means and 95% confidence limits from the three repeats for all 12 conditions were determined and statistical analysis were completed using two‐way ANOVA with significance taken at *p* < .05.

(1) $$Severity\;of\;Edge\;Loading=\int_{t0}^{t}F\left(x\right).dt+\int_{t0}^{t}F\left(y\right).dt$$

where *F*(*x*) is the medial–lateral, *F*(*y*) is the axial load, and *t*_0_–*t* is the duration of edge loading.

Six of the 12 conditions used in stage one were chosen to further assess the severity of edge loading and station variation with a larger sample number (*n* = 6). The conditions were the combinations of 45° and 65° acetabular cup inclination angles and 2, 3, and 4 (mm) medial–lateral translational mismatch.

These six conditions were then used to assess the wear performance (third stage of the study) of ceramic‐on‐ceramic bearings. Each wear test ran for a total of three million cycles and six repeats were completed for the six chosen conditions. Gravimetric analyses of the femoral heads and acetabular cups were completed before the testing commenced and at one million cycle intervals. The gravimetric analysis was done using a micro balance (Mettler‐Toledo XP205, UK) which had a readability of 0.01 mg. The components were cleaned in a consistent manner according to internal protocols and were acclimatised in a temperature and humidity controlled environment in the balance room prior to weighing. The wear rate was calculated from the difference in weights measured at every measurement point and converted into volume by multiplying by the density of the ceramic material (0.0044 g/mm^3^). The mean values for severity of edge loading and wear rates with 95% confidence limits were determined and Student's *t* test was used as a statistical analysis with significance taken at *p* < .05.

The femoral heads were measured geometrically at the end of the three million cycles using a coordinate measuring machine (CMM, Mitutoyo, Legex 322, Japan). Over 9,000 data points were collected on the surface of each the femoral head and the data cloud were exported to RedLux software (RedLux, Southampton, UK) to plot a three‐dimensional representations of the wear area. The location, orientation, size, and depth of the wear scar were determined. Surface roughness was measured over the wear area on the femoral heads using a two dimensional contacting profilometry (Talysurf PGI 800, Taylor Hobson, UK) and compared to pretest roughness values. The mean *R*_a_ was determined for all femoral heads and Student's *t* test was used to determine if a significant increase in roughness was obtained under each condition. The microstructure of the damage caused under severe edge loading was observed using a scanning electron microscope (SEM, Carl Zeiss EVO MA15, Germany).

## RESULTS

**Figure 4:**
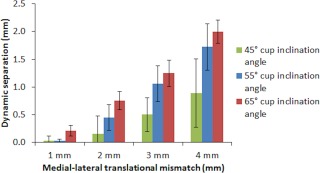
Mean magnitude of dynamic separation ±95% confidence limits (*n* = 3) at different surgical positions including acetabular cup and femoral head translational mismatch and acetabular cup inclination angles.

**Figure 5:**
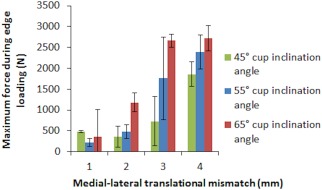
Mean maximum load recorded at the rim during edge loading ±95% confidence limits (*n* = 3) at different surgical positions including acetabular cup and femoral head translational mismatch and acetabular cup inclination angles.

**Figure 6:**
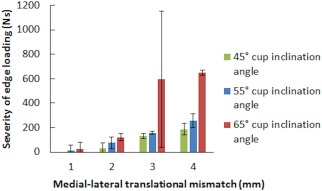
Mean severity of edge loading ±95% confidence limits (*n* = 3) at different surgical positions including acetabular cup and femoral head translational mismatch and acetabular cup inclination angles.

The results from the biomechanical test (stage one of this study) demonstrated that as the input translational mismatch increased from 1 to 2 mm to 3 to 4 mm, the magnitude of dynamic separation, the maximum load recorded at the rim during edge loading and the severity of edge loading increased (Figures 4, 5, 6, respectively). This increase was significant for all groups for the dynamic separation maximum load at the rim and, for the severity of edge loading (*p* < .01). The levels of dynamic separation recorded were in excess of 0.5 mm (the level fixed in the previous studies) in half (six) of the position configurations considered in this study. There was no significant difference in the severity of edge loading between 1 and 2 mm and between 3 and 4 mm translational mismatch conditions. Furthermore, as the acetabular cup inclination angle increased from 45° to 55° to 65°, the magnitude of the dynamic separation displacement, the maximum load recorded at the rim and the severity of edge loading also increased (Figures 4, 5, 6, respectively).There was no significant difference (*p* = .38) in the severity of edge loading between the 45° and 55° cup inclination angle. However, increasing the inclination angle to 65° caused significant (*p* < .01) increase in severity of edge loading.

**Figure 7:**
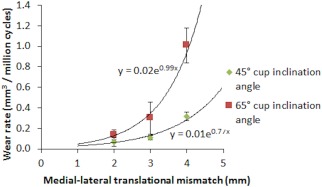
Mean wear rate ±95% confidence limits (*n* = 6) of ceramic‐on‐ceramic bearings against the initial medial–lateral translational mismatch of the centres of the femoral heads and acetabular cups under the six different conditions.

**Figure 8:**
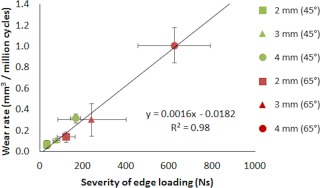
Mean wear rate ±95% confidence limits (*n* = 6) of ceramic‐on‐ceramic bearings against the mean severity of edge loading ±95% confidence limits (*n* = 6).

Stage three of this study (the wear study) showed that increasing the translational mismatch of components' centres of rotation from 2 to 3 to 4 (mm) resulted in an increased wear rate of ceramic‐on‐ceramic bearings for both the cup inclination angles (Figure 7), with the 65° cup inclination angle having significantly higher wear rate than the cup inclination angle condition of 45° (*p* = .02, *p* = .02, and *p* < .01, respectively). The mean wear rates (±95% CL) for the 2, 3, and 4 mm translational mismatch conditions with the cup inclination angle at 45° were 0.07 ± 0.04, 0.11 ± 0.02, and 0.32 ± 0.04 mm³/million cycles, respectively. The mean wear rates for the 2, 3, and 4 mm conditions with the cup inclination angle at 65° were 0.14 ± 0.05, 0.30 ± 0.16, and 1.01 ± 0.17 mm³/million cycles, respectively. The wear rate was found to correlate positively (*R* ^2 ^= 0.98) with the severity of edge loading for the 45° and 65° cup inclination angles (Figure 8). Large variation in the severity of edge loading between the three repeats was obtained under the 65° cup inclination angle and 3 mm translational mismatch condition (Figure 6). This level of positioning appeared to lie on a threshold at which the severity of edge loading significantly increased.

**Figure 9:**
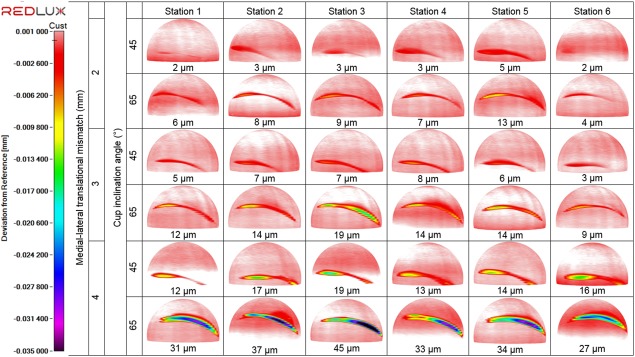
Three dimensional reconstruction of the femoral heads showing the location, orientation and depth of the stripe wear formed on each of the ceramic femoral heads under the different testing conditions of 2, 3, and 4 mm of translational mismatch in the centres of the femoral head and acetabular cup and two inclination angles of 45° and 65° (*n* = 6).

**Figure 10:**
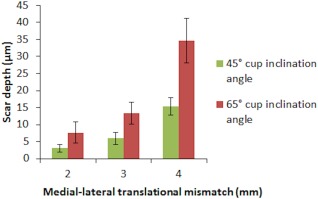
The mean penetration depth ±95% confidence limits (*n* =  6) of the wear stripe on all femoral heads under the different testing conditions of 2, 3, and 4 mm of translational mismatch in the centres of the femoral head and acetabular cup and two inclination angles of 45° and 65°.

**Figure 11:**
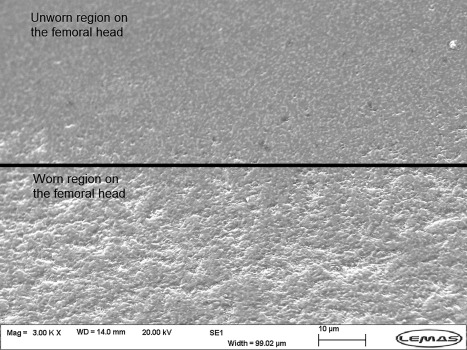
Scanning electron microscope image of the wear stripe on the femoral head at three million cycles of testing under 65° cup inclination angle and 4 mm translational component mismatch.

Under all six conditions studied in the wear tests, stripe wear, a consequence of edge loading, was observed visually without any aid on all femoral heads with a corresponding wear area at the rim of the acetabular cups. However, the severity of the stripe wear was difficult to quantify without the CMM. The orientation, location, and depth of the wear stripe varied on the femoral heads under the different conditions investigated (Figure 9). The penetration depths of the wear stripe significantly increased as the level of dynamic separation and the severity of edge loading increased (*p* < .01, Figure 10). The combination of steep inclination angle with large level of translational surgical mismatch caused the femoral head to stay on the edge of the acetabular cup longer during the gait cycle. This resulted in a more severe edge loading condition leading to a deeper wear area. In some instances, squeaking originating from the bearings was also heard under these severe conditions. This observation was more prevalent and persistent under the 65° cup inclination angle with 4 mm translational mismatch of the components centres of rotation. Under all edge loading conditions, the mean surface roughness *R*_a_, over the wear stripe significantly increased (Table 1, *p* < .01) and SEM images showed grain removal at the surface (Figure 11).

**Table 1:** Mean Roughness Ra for Unworn and Worn Ceramic Femoral Head Components Under All Conditions Tested in Stage Three of the Study

	Unworn	Medial–Lateral Translational Mismatch (mm)					
		2		3		4	
		Cup Inclination Angle (°)					
		45	65	45	65	45	65
Mean *R*_a_ (µm)	0.006	0.012	0.011	0.016	0.018	0.019	0.018
95% confidence limit	0.001	0.002	0.002	0.004	0.007	0.003	0.007

## DISCUSSION

Stripe wear on retrieved ceramic femoral heads was reported by Nevelos et al.^1^ This wear mechanism was not replicated *in vitro* when hip bearings were tested under the standard simulator condition where the centres of rotation of the femoral head and acetabular cup were concentric. Steep cup inclination angle conditions did not cause the formation of stripe wear on the femoral head.^27^ Nevelos et al.^11^ modified their simulator protocol to apply a mismatch between the centre of rotation of the femoral head and the acetabular cup causing sliding motion between the femoral head and acetabular cup in the medial–lateral axis, a methodology termed “microseparation”. Under this methodology, the acetabular cup moved medially and superiorly relative to the femoral head during the swing phase of the gait cycle without losing contact causing edge loading at heel strike. Nevelos et al.^11^ and subsequent studies investigating the effect of edge loading due to microseparation conditions on the wear of different bearing combinations used a fixed level of microseparation of approximately 500 µm in the medial–lateral axis. This methodology, although yielding useful data, has the limitation that it only determined the effect of a predetermined level of (microseparation) edge loading on the wear. It did not provide information on the likelihood or the magnitude of its occurrence associated with variations in position or design. In this study however, the input to the experimental setup was the surgical mismatch between the centres of rotation of the femoral head and acetabular cup in the medial–lateral axis and the rotational (inclination) positioning. The outputs of the study were the dynamic microseparation as well as the load acting on the rim and the wear. Under some conditions, the resultant dynamic separation between the femoral head and acetabular cup was in excess of 1 mm thus requiring us to refer to this condition in this study as “separation” and not “microseparation”. It is also important to highlight that no loss of contact between the femoral head and acetabular cup occurred under any of the conditions applied and the separation referred to, is the separation between the centres of rotation of the femoral head and the cup as the head slid over the rim of the acetabular cup. There is no precise clinical data relating the effect of the surgical offset on the medial–lateral force during the swing phase load. Indeed, the magnitude of the medial–lateral force during the swing phase when the bearing is concentric is subject to considerable variation and uncertainty. A 100 N/mm spring was chosen to compare with previous studies,^11^ where wear and damage had been compared to retrievals in ceramic‐on‐ceramic bearings.

Under this newly developed simulation method, the occurrence and severity of edge loading can be determined under a wide range of clinically relevant conditions (surgical positions) and each condition can be investigated in isolation or in combination with other factors. These factors can be the orientation of the cup (inclination, version, and tilt), the mismatch between the centres of rotation of the cup and head in medial–lateral, anterior–posterior, and superior–inferior directions, the variations in soft tissue tension, the different kinetics and kinematics of the patients and implant designs. However, in the current study, only the variations in inclination angle and mismatch between the centres of rotation of the femoral head and acetabular cup in the medial–lateral axis were considered.

The level of mismatch between the centre of rotation of the femoral head and acetabular cup increases the severity of edge loading and this increase is indicated by the level of dynamic separation, time the head spent on the rim during the gait cycle and also the load reached while the head was still on the rim of the acetabular cup. Higher levels of dynamic separation resulted in higher loads at the rim. This will lead to higher stress under edge loading^22^ conditions contributing to material fatigue and higher wear.

**Figure 12:**
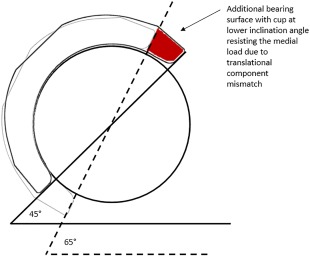
Schematic showing the acetabular cup at two inclination angle conditions highlighting the additional bearing surface with the cup at lower inclination angle resisting the medial load due to translational component mismatch.

Under translational mismatch, increasing the inclination angle also resulted in significant increase in the medial–lateral separation displacement and the wear of ceramic‐on‐ceramic bearings highlighting the benefits of avoiding steep cup inclination. The cup positioned at a lower inclination angle of 45° resisted the resultant medial motion caused by the mismatch between the centres of rotation of the head and the cup, better than that of the cups positioned at 65° due to the additional bearing surface that the load has to overcome as illustrated in Figure 12, resulting in lower levels of dynamic separation.

All conditions in this study with a mismatch of the head and cup centres of 2 mm or greater generated edge loading and wear stripe on all femoral heads, and a corresponding wear area on the rim of the cups. The position and orientation of the highest penetration in the wear stripe changed with different rotational and translational positions. A greater wear scar depth and stripe wear area were measured with increased mismatch in the head and cup centres of rotations. The severity of edge loading and damage was greater in this study than that of previous tests,^18^ and some severely damaged areas were detected with the SEM.

Surface roughness measurements only indicated an increase in roughness over the wear area under edge loading. It could be possible that the areas of severe wear were masked as it was difficult to completely evaluate the stripe wear when taking single traces.

During gait, the hip joint forces act in all three axes, so the direction in which the head translates with respect to the cup will depend on the patients' biomechanics as well as the implant position. Different patient activities also result in varying directions of applied load and motions,^44^ thus will present different scenarios of edge loading. Future studies will also consider variations in the swing phase load as well as the effect of different spring rates. In surgery, translational and rotational positioning can vary in six degrees of freedom. In this study a standard gait cycle was applied and only the variation of two degrees of freedom were considered. Other conditions may also lead to dynamic separation and edge loading such as translational mismatch in the anterior–posterior and superior–inferior axis and both version and tilt of the cup. These will be studied in the future as more variables are added to the experimental system.

The translational surgical variations of the head and cup centres that occur *in vivo* are very difficult to measure and identify. However the levels of dynamic separation during gait that have been recorded using fluoroscopy studies^8^, ^10^, ^45^, ^46^ reported a significant patient to patient variation which fell in the range simulated in this study.

This study only tested and compared ceramic‐on‐ceramic bearings, but the same principle should apply to all hard‐on‐hard bearings, thus it can provide an explanation for the higher than expected wear rates which have resulted in high revision rates of metal‐on‐metal bearings. Regarding hard‐on‐soft (that is, polyethylene) hip replacement, rim cracking has been observed in retrievals,^47^ thus rim loading can occur and may lead to fatigue of the material as it was loaded against the metal backing. This new simulation methodology can predict the severity of edge loading and potential consequences for fatigue life as a function of surgical position. Furthermore, polyethylene liners, unlike liners used in hard‐on‐hard bearings frequently have an extended superior lip to improve stability and resistance to dislocation. This methodology can be used in the future to assess the relative benefits of such design changes.

Evidence of edge loading is frequently observed in retrievals. Earlier methodologies could only indicate the effect of a fixed predetermined level of microseparation and edge loading. This study shows clearly the occurrence and severity of edge loading is highly dependent on the component positioning and conditions used for testing in a hip joint simulator. The relationships between the variation in component positioning and severity of edge loading and wear have been described for the first time. The approach used in this study can now be adopted to advance and enhance preclinical testing of hip prostheses.

## CONCLUSION

An advanced physiological *in vitro* simulator method, that can predict the occurrence and severity of edge loading and the wear of different hip replacements made from different materials and designs due to variations in component positioning, developed in this study, can be used as a preclinical testing technique to better predict the efficacy and reliability of new hip replacement bearings. This study demonstrated how variations in rotational and translational component positioning affect the occurrence and severity of edge loading under a set of kinematic conditions in a hip joint simulator. It provides an indication which supports the rationale for aligning the head and cup centres and correctly positioning the cup inclination angle during total hip joint replacement.

Lower wear was found with the cup inclination angles at 45° which showed greater resistance to dynamic separation as a result of surgical joint centre mismatch in the medial–lateral axis under a set of kinematic conditions. When a higher mismatch was employed, the level of dynamic separation increased, and thus the wear. This study suggests that optimal surgical position should not only consider the rotational position of the acetabular cup but also the relative positions of the centres of rotation of the femoral head and the acetabular cup.
